# Rare *CACNA1H* and *RELN* variants interact through mTORC1 pathway in oligogenic autism spectrum disorder

**DOI:** 10.1038/s41398-022-01997-9

**Published:** 2022-06-06

**Authors:** André Luíz Teles e Silva, Talita Glaser, Karina Griesi-Oliveira, Juliana Corrêa-Velloso, Jaqueline Yu Ting Wang, Gabriele da Silva Campos, Henning Ulrich, Andrea Balan, Mehdi Zarrei, Edward J. Higginbotham, Stephen W. Scherer, Maria Rita Passos-Bueno, Andrea Laurato Sertié

**Affiliations:** 1 Hospital Israelita Albert Einstein, São Paulo, Brazil; 2 Centro de Estudos do Genoma Humano e Células Tronco, Instituto de Biociências, Universidade de São Paulo, São Paulo, Brazil; 3 Instituto de Química, Universidade de São Paulo, São Paulo, Brazil; 4 Instituto de Ciências Biomédicas, Universidade de São Paulo, São Paulo, Brazil; 5 The Centre for Applied Genomics, Genetics and Genome Biology, The Hospital for Sick Children, Toronto, ON Canada; 6 Department of Molecular Genetics, University of Toronto, Toronto, ON Canada

**Keywords:** Medical genetics, Autism spectrum disorders, Stem cells

## Abstract

Oligogenic inheritance of autism spectrum disorder (ASD) has been supported by several studies. However, little is known about how the risk variants interact and converge on causative neurobiological pathways. We identified in an ASD proband deleterious compound heterozygous missense variants in the Reelin (*RELN*) gene, and a *de novo* splicing variant in the Cav3.2 calcium channel (*CACNA1H*) gene. Here, by using iPSC-derived neural progenitor cells (NPCs) and a heterologous expression system, we show that the variant in Cav3.2 leads to increased calcium influx into cells, which overactivates mTORC1 pathway and, consequently, further exacerbates the impairment of Reelin signaling. Also, we show that Cav3.2/mTORC1 overactivation induces proliferation of NPCs and that both mutant Cav3.2 and Reelin cause abnormal migration of these cells. Finally, analysis of the sequencing data from two ASD cohorts—a Brazilian cohort of 861 samples, 291 with ASD; the MSSNG cohort of 11,181 samples, 5,102 with ASD—revealed that the co-occurrence of risk variants in both alleles of Reelin pathway genes and in one allele of calcium channel genes confer significant liability for ASD. Our results support the notion that genes with co-occurring deleterious variants tend to have interconnected pathways underlying oligogenic forms of ASD.

## Introduction

Large-scale genomic analyses have revealed that the genetic susceptibility to autism spectrum disorder (ASD) is shaped by rare and common variants and varies from one individual to another. While in a few cases a single rare genetic variant with large effect appears sufficient to cause ASD [1–4], in most cases the genetic landscape of the disease is much more complex and involves thousands of common risk alleles, individually of small effect but collectively conferring susceptibility above a threshold of liability [5–8]. In addition, in several individuals with ASD the clinical outcome seem to be the result of the joint combination of more than one rare variant with reduced penetrance in an oligogenic (≥2 hits) mode of inheritance [9–13]. Therefore, a key step in understanding the neurobiological basis of ASD is to decipher the genetic interactions between ASD-risk variants. However, the additive or epistatic interactions of these risk alleles are still largely unexplored.

Recently, we have identified in one Brazilian individual with autism and macrocephaly rare compound heterozygous missense variants in the *RELN* gene and a *de novo* splice site variant in the *CACNA1H* gene [14–16]. The *RELN* gene encodes Reelin, a large secreted glycoprotein that controls neuronal migration and plasticity of synapses [17, 18]. The *CACNA1H* gene encodes the α1-subunit of the T-type low voltage-dependent calcium (Ca^2+^) channel Cav3.2, known to control neuronal excitability [19, 20]. Although rare variants have been identified in *RELN* and *CACNA1H* genes in ASD individuals, several of them did not segregate with the disease in the affected families and seem to require additional genetic, epigenetic or environmental factors to cause the clinical phenotype [1, 21–23].

We have previously shown that the variants in *RELN* identified in the Brazilian proband are deleterious and lead to diminished Reelin secretion and impaired Reelin signal transduction in induced pluripotent stem cells (iPSC)-derived neural progenitor cells (NPCs). Also, our results suggested that mTORC1 signaling is overactivated in *RELN* mutant NPCs and contributes to the downregulation of the Reelin cascade [14]. However, the underlying molecular mechanism of mTORC1 pathway hyperfunction remains unknown. Because mTORC1 pathway can be regulated by intracellular Ca^2+^ levels [24, 25], in this study we investigated whether the variant identified in *CACNA1H* in the Brazilian proband functions as a ‘second hit’, leading to changes in Ca^2+^ influx and contributing to overactivation of the mTORC1 pathway and to downregulation of the Reelin signaling in NPCs, a cell population that endogenously express low-voltage-activated Ca^2+^ channels [16, 26, 27], and that can reveal important clues about both early stages of brain development and ASD pathophysiology [14, 16, 28–30]. We also investigated whether the *CACNA1H* and *RELN* variants cause abnormal NPC phenotypes. Finally, we examined whether the co-occurrence of damaging variants in genes for the Reelin cascade and Ca^2+^ channels may also be observed in other ASD individuals using sequencing data from different ASD cohorts.

## Methods

### Subjects and genetic analysis

This study was approved by the Ethics Committees of the Instituto de Biociências da Universidade de São Paulo and of the Hospital Israelita Albert Einstein, as well as by the Western Institutional Review Board, Montreal Children’s Hospital–McGill University Health Center Research Ethics Board, McMaster University–Hamilton Integrated Research Ethics Board, Eastern Health Research Ethics Board, Holland Bloorview Research Ethics Board and The Hospital for Sick Children Research Ethics Board. Informed consent was obtained from all participants. All individuals whose cells were evaluated in this study were described previously [14–16]. Whole-exome sequencing (WES) was performed using genomic DNA from peripheral blood of 861 Brazilian individuals from 279 trio and 6 quartet families of children diagnosed with ASD (*n* = 291 individuals with ASD, 570 unaffected parents) and analyzed as described previously [4, 14–16]. By using a custom script, WES data was subsequently re-analyzed for the presence of co-occurring rare (Global MAF ≤ 0.01 in gnomAD, 1000G, ESP6500, and AbraOM) and predicted impactful (CADD score ≥20) variants in both alleles of Reelin pathway genes and in at least one allele of Ca^2+^ channel genes (Supplementary Table S1). The findings were validated using whole-genome sequencing (WGS) data from the MSSNG cohort [3] (*n* = 4258 families of children with ASD; 11,181 samples, 5102 with ASD) and the same selection criteria. For details in WES and WGS and data processing see Supplementary information. The selected variants in Reelin pathway and Ca^2+^ channel genes (Table 1) were subsequently reanalyzed with 13 additional pathogenic prediction tools (Supplementary Table S2) and were validated by Sanger sequencing. Clinical characteristics of the 10 individuals with ASD from both cohorts who harbor these variants are described in Supplementary Table S3.

### Three-dimensional modeling

The 3D models of wild-type and mutant Cav3.2 channels were built using the online I-Tasser server [31]. Three sets of models for each protein were built using the amino acids corresponding to domains I–II, II–III, and III–IV. The motifs were then superimposed onto the crystal structure coordinates of the complex of voltage-gated sodium channel NavPaS from the American cockroach Periplaneta americana (PDB code 6A91) using the secondary-structure matching tool [32] and Coot v.0.8.2 [33]. Pymol [34] was used to create the structural figures. Protein hydrophobicity plots were produced using Expasy Protscale [35].

### Neural progenitor cells culture

All NPC samples used in this study have been previously differentiated from iPSCs and were cultured as described previously [14, 16]. NPCs derived from 2 iPSC clones of the Brazilian patient harboring the rare variants in the *RELN* and *CACNA1H* genes (referred to as F2688-1), and either 1 or 2 iPSC clones of the other subjects (*n* = 4 control individuals) were used in all experiments described herein.

### Cloning of the mutated *CACNA1H* and HEK293T transfection

cDNA samples from patient F2688-1 were used to amplify by conventional PCR the region of the *CACNA1H* gene containing the 156 bp insertion (intron 13). The purified PCR fragment was then introduced into the wild-type human Cav3.2 cDNA (plasmid a1Ha-pcDNA3, #45809 Addgene) between exons 13 and 14 using standards molecular cloning techniques. The presence of the mutation and integrity of the construct were verified by Sanger sequencing. HEK293T cells were transfected with either wild-type or mutated a1Ha-pcDNA3, or empty pcDNA3 vector (negative control) using the Lipofectamine™ 3000 Transfection Reagent (ThermoFisher). RNA and protein were extracted from cells 48 h after transfection. The overexpression of WT- and MUT-a1Ha was confirmed by quantitative reverse transcription-PCR **(**RT-qPCR), which showed very similar levels of expression (data not shown).

### Conventional and quantitative reverse transcription-PCR analyses

Extraction of total RNA from each cell sample and cDNA synthesis was performed as described previously [14]. Conventional PCR reactions were performed with a forward primer designed to anneal in exon 12 and a reverse primer designed to anneal in exon 15 of *CACNA1H*. Sanger sequencing was carried out to confirm intron 13 retention in the mature *CACNA1H* transcript. Quantitative PCR (qPCR) reactions were performed with predesigned TaqMan gene expression assays (ThermoFisher), and *HMBS* as a housekeeping gene. All qPCR samples were run in triplicate. The comparative CT method (^ΔΔ^CT Method) was used to analyze the expression levels of *CACNA1H*. The experiments were repeated twice with similar results.

### Cell treatments

The cells were cultured in the presence of vehicle (DMSO) or 10 μM of the T-type Ca^2+^ channels blocker NNC 55-0396 dihydrochloride (Tocrisis Bioscience) for 30 min prior to protein extraction for western blot analysis or measurements of extracellular Ca^2+^ influx. For the proliferation and migration assays, NPCs were cultured in the presence of vehicle (DMSO), 1 uM of NNC 55-0396 dihydrochloride, 100 nM of rapamycin (Sigma Aldrich), wild-type Reelin- or mock-conditioned medium (prepared as previously described [14]) during the entire course of the experiments.

### Measurement of extracellular Ca^2+^ influx

The fluorescence-based FLIPR^®^ Calcium 4 Assay Kit (Molecular Devices) was used to detect changes in intracellular Ca^2+^. Briefly, the cells (3–5 × 10^4^ cells/well) were seeded in black 96-well plates, and cultured in growth medium for 48 h. Before initiating the assay, the medium was removed and cells were incubated with the Ca^2+^ dye containing 2.5 mM probenecid for 60 min at 37 °C. The cells were then treated with vehicle or NNC 55-0396 dihydrochloride, and the dye was excited at 480 nm and its fluorescence signals detected at 525 nM. The basal fluorescence intensity was monitored for 15 s at 1.65 s intervals, and then cells were challenged with 100 mM KCl and the cellular fluorescence continued to be recorded over 200 s. KCl-evoked increases in intracellular Ca^2+^ were determined as Δ*F*/*F*0 (*F*0 is basal fluorescence) using the SoftMax2Pro software (Molecular Devices Corp.). The Δ*F*/*F*0 values obtained from the same group did not differ significantly at either time points after KCl stimulation. Each experimental condition was performed in three replicate wells for NPCs and five replicates wells for HEK293T cells. The experiments were repeated at least twice with similar results.

### Protein extraction and immunoblotting

Extraction of total proteins from cell samples, standard western blotting, and quantification of band intensity were carried out as described previously [14]. The anti-DAB1, anti-pRPS6, anti-pSRC, anti-SRC, and anti-ßactin antibodies used are described in [14], and results shown are from two independent experiments. In addition, the following primary antibodies were used in order to detect endogenous Cav3.2 protein in NPCs and other cell types (including Hela and HEK293T cells transfected with WT-a1Ha) but, despite several attempts, neither of them worked properly: ab135974 and ab128251 (Abcam), sc-377510 (Santa Cruz Biotechnology).

### Analysis of cell morphology, size, and proliferation

NPC samples were seeded into 12-well plates at density of 1.0 × 10^5^ cells per well and cultured in complete NPC medium for 24 h. Phase-contrast images were obtained at 20× magnification using the IncuCyte® system (Essen Bioscience). Analysis of cell morphology and measurements of cell body sizes (including soma size and all cell surface projections) were performed using the IncuCyte Neurotrack software. For the proliferation assay, 24 h after initial plating (Day 0), cells were counted at 48 h and 72 h using trypan blue exclusion and automated cell counting. Each experimental condition was performed in 2 replicate wells. The experiments were repeated at least twice with similar results.

### Analysis of cell migration (scratch-wound healing assay)

NPC samples were seeded into 96-well plates at density of 3.5 × 10^4^ cells per well and cultured in complete NPC medium until 90% confluence. The cells were then grown in DMEM/F12 without FGF and EGF for 48 h to inhibit cell proliferation, and the 96-well WoundMaker (Essen Bioscience) was used to generate a wound area in the confluent monolayer of cells. Wound closure was monitored and quantified with the IncuCyte® system (Essen Bioscience). Relative wound density was defined as cell density in the wound area expressed relative to the cell density outside of the wound area over time. Each experimental condition was performed in 6 replicate wells. The experiments were repeated at least twice with similar results.

### Statistical analysis

Statistical analyses were conducted using SPSS statistical software (IBM SPSS Statistics 20). The generalized linear mixed-effect models were used to account for dependency between biological replicates (when NPCs derived from different iPSC clones of the same individual were used) and independent technical replicates per individual, and to investigate association between the response and the explanatory variables (time period and treatments). Fisher’s Exact Test was used to determine if there is an enrichment of co-occurring rare variants in Reelin pathway and Ca^2+^ channel genes in ASD individuals. The PASS (Power Analysis and Sample Size) software was used to calculate statistical power, and the results showed that the sample size ensured a statistical power ≥0.80 for most analyses in this study. Differences were considered significant when Bonferroni-corrected *p* < 0.05. Data are presented as medians with interquartile ranges.

## Results

### The *CACNA1H* splice site variant identified in patient F2688-1 causes intron retention in the mature transcript

Through WES, we have identified in one individual with ASD and macrocephaly, referred to as F2688-1, the previously characterized compound heterozygous missense variants in the *RELN* gene [14], and a *de novo* heterozygous splice donor site variant in intron 13 of the *CACNA1H* gene (NM_001005407:c.2907+1 G>A) (Table 1; Supplementary Table S2; Supplementary Fig. S1A). This variant was absent from the population databases, and is predicted to inactivate the 5′ splice site of intron 13, which is only 156 bp long, causing the retention of this intron in the mature *CACNA1H* mRNA. Because the retained intron does not cause a frameshift in the *CACNA1H* coding sequence, it is expected to introduce an in-frame insertion of 52 novel amino acid residues in the pore-forming region of the Cav3.2 channel.

**Fig. 1:**
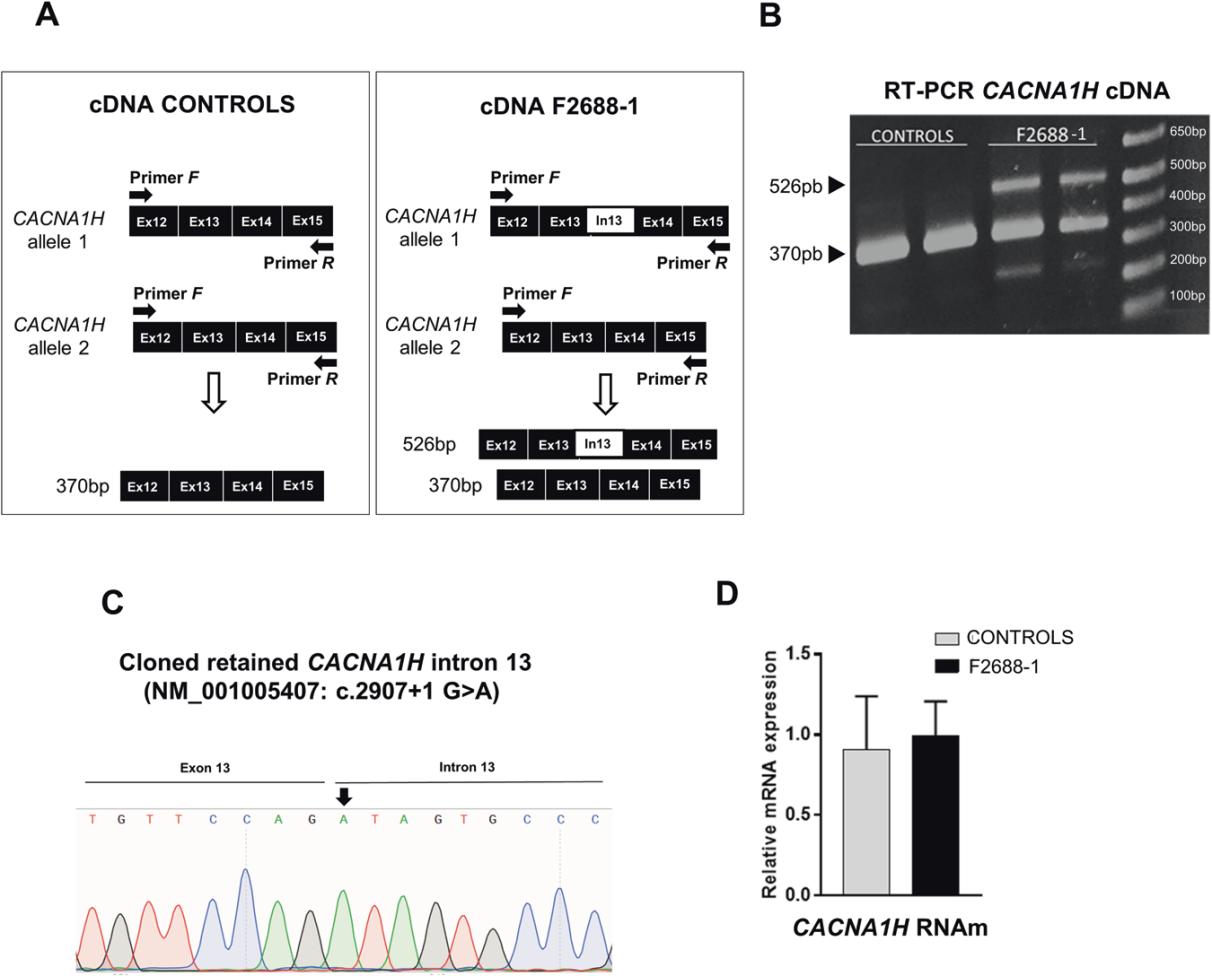
Splice donor site variant in intron 13 of the *CACNA1H* gene in patient F2688-1 results in retention of the entire intron in the mature mRNA. **A** Schematic representation of intron 13 (In13) retention in the *CACNA1H* transcript showing the locations of primers for conventional RT-PCR. The PCR band corresponding to the wild-type allele is 370 bp long, whereas the band corresponding to the mutant allele is 526 pb long due to retention of intron 13 in the mature transcript. **B** Representative image of agarose gel electrophoresis showing conventional RT-PCR products. The 526 bp PCR product of the mutant allele is only observed in F2688-1-derived NPCs (*n* = 2 iPSC clones-derived NPCs). **C** Sanger sequencing of the cloned 526 bp PCR band confirmed intron 13 retention in the mature *CACNA1H* transcript. The location of the variant NM_001005407:c.2907+1 G>A is shown. **D** Quantitative RT-PCR demonstrated that the relative transcription levels of *CACNA1H* are not changed in F2688-1-derived NPCs (*n* = 2 iPSC clones-derived NPCs) compared with control NPCs (Controls, *n* = 4). The bar graph shows the median value and interquartile range for each group.

We, therefore, sought to investigate whether the removal of intron 13 from the pre-mRNA of *CACNA1H* was abnormal in neural cells derived from patient F2688-1 using cDNA samples from iPSC-derived NPCs from this patient and from control individuals. We observed, as expected, that the variant causes abnormal splicing and intron 13 retention in the mature *CACNA1H* transcript (Fig. 1A–C). Despite the defective RNA splicing, we observed no significant change in the transcript levels of *CACNA1H* in F2688-1 NPCs compared to control NPCs (Fig. 1D).

### Structural modeling of the mutant Cav3.2 channel suggests a larger pore diameter than the wild-type channel

**Fig. 2:**
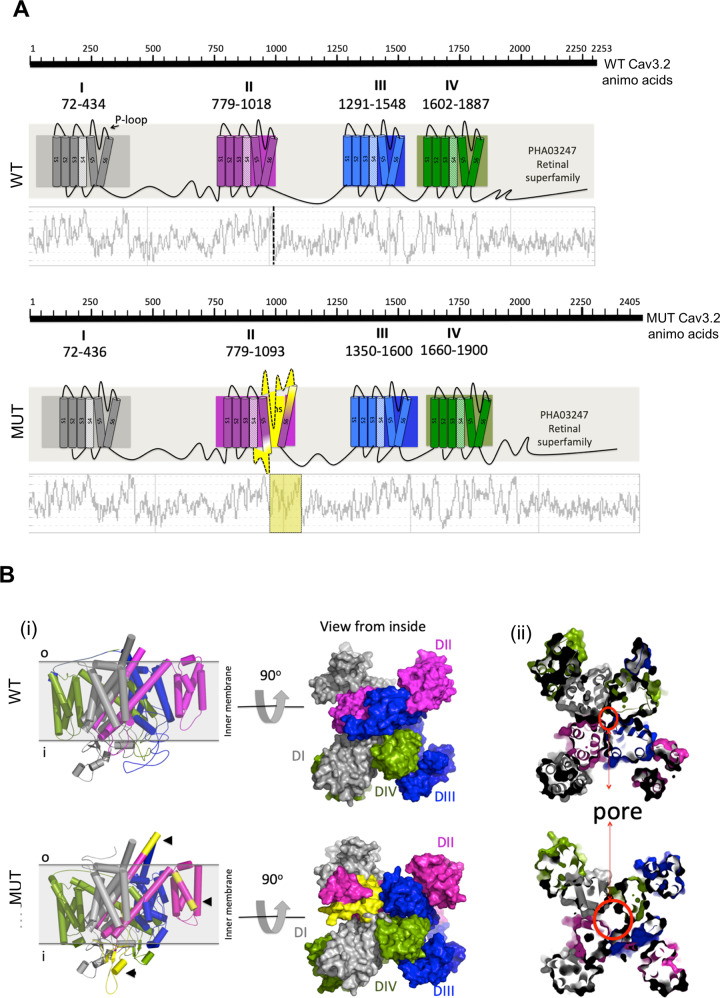
3D model of the mutant Cav3.2 channel reveals significant structural changes. **A** Predicted topologies of the wild type (WT) and mutant (MUT) Cav3.2 channels showing the four homologous domains (I–IV) in different colors, each composed of six transmembrane segments (bars S1–S6). The 52-amino acid insertion (yellow) is located between segments S5 and S6 of domain II (between residues Q^969^ and I^970^). The hydropathy profiles of the amino acid sequences are shown below the channel topologies. **B** Structural features of the WT and MUT Cav3.2 channels. **Bi** Simulations of the proteins viewed from the cytosolic side of the plasma membrane are shown in cartoon representation and colored by domain (DI–DIV). Domain II is colored in magenta and the 52-amino acid insertion is detached in yellow. **Bii** Structural view of the pore center showing the differences in the pore diameter between WT and MUT Cav3.2 channels.

Cav3.2 channel consists of four homologous domains (I–IV), each one composed of six α-helical transmembrane segments (S1–S6). The voltage-sensing module of the channel is formed by the positively charged S4 segments, while the ion conductivity and selectivity lie on the negatively charged extracellular membrane-reentrant pore loop (P-loop) between S5 and S6 [20, 36] (Fig. 2A). We observed that the 52-amino acid insertion, located between segments S5 and S6 of domain II, alters the hydropathy profile in the pore region and causes significant conformational changes in the next domain (Fig. 2A, yellow). To evaluate the impact of the insertion on the Cav3.2 channel structure, we built 3D models of the wild-type and mutant proteins. We observed, as pointed by the domain and hydropathy profile analyses, that the insertion (Fig. 2Bi, yellow) alters the secondary structure of domain II (Fig. 2Bi, magenta) and also impacts the folding of domain III (Fig. 2Bi, blue), changing the position of transmembrane helices that compose the pore-forming domain and increasing the pore diameter in the mutant protein (Fig. 2Bii). Together, these analyses suggest that the insertion induces significant structural changes in the mutant channel, resulting in larger open pores.

### Calcium influx through the mutant Cav3.2 channel is increased and induces exaggerated mTORC1 signaling in F2688-1 neural progenitor cells

**Fig. 3:**
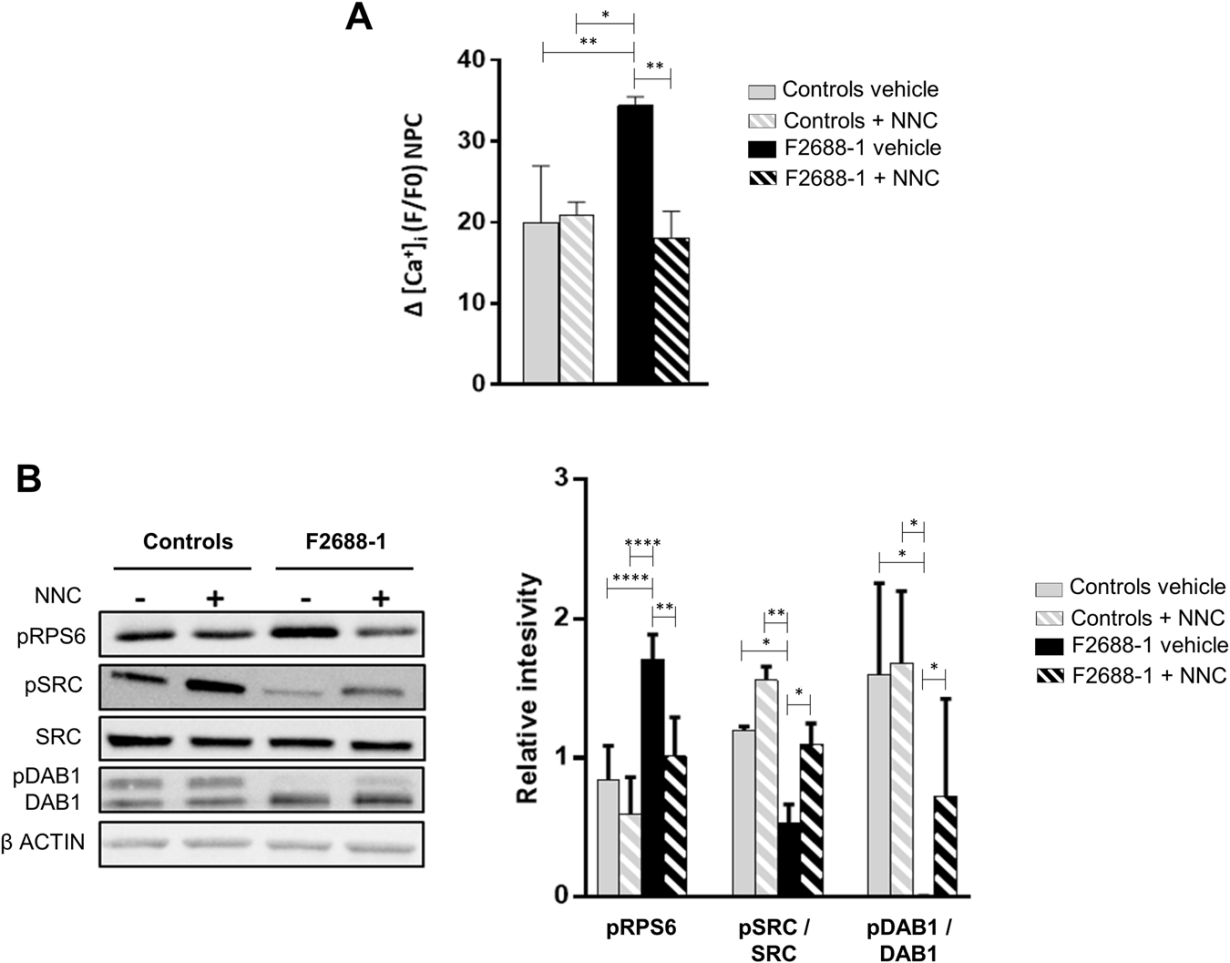
Increased Ca^2+^ influx through the mutant Cav3.2 channel causes exaggerated mTORC1 signaling in F2688-1-derived NPCs, which, in turn, contributes to impaired Reelin signaling in these cells. **A** Intracellular Ca^2+^ measurements in control-derived NPCs (*n* = 4) and in F2688-1-derived NPCs (NPCs derived from 2 iPSC clones) depolarized with 100 mM KCl and cultured in the absence (vehicle) or presence of 10 μM of the T-type Ca^2+^ channels blocker NNC 55-0396 (NNC). The bar graph shows the median value and interquartile range for each group. F2688-1 NPCs show significantly increased Ca^2+^ influx compared with control NPCs, which was rescued by treatment with NNC. **B** Representative immunoblots showing the expression levels of pRPS6, pSRC, SRC, pDAB1, DAB1, and β-actin (used as loading control) in control-derived NPCs (*n* = 4) and in F2688-1-derived NPCs (NPCs derived from 2 iPSC clones) cultured in the absence (−) or presence (+) of 10uM of NNC. The bar graph shows the median levels and interquartile ranges of normalized pRPS6, pSRC, and pDAB1 for each group. While F2688-1-derived NPCs cultured in the absence of NNC show elevated levels of pRPS6 and diminished levels of pSRC and pDAB1, treatment with NNC significantly rescued pRPS6 and pSRC expression to levels similar to the untreated control NPCs. On the other hand, while treatment with NNC significantly improved pDab1 levels in F2688-1 NPCs, there is still a clear trend towards diminished expression of pDAB1 in these cells compared to untreated control cells. *p < 0.05, **p ≤ 0.01, ****p ≤ 0.0001.

To assess the functional impact of the 52-amino acid insertion on Cav3.2 channel activity, we measured the influx of extracellular Ca^2+^ in F2688-1 and control-derived NPCs. We found that F2688-1 NPCs show significantly increased Ca^2+^ influx after depolarization compared with control NPCs, which was abolished by treatment with the T-type Ca^2+^ channels blocker NNC 55-0396 (Fig. 3A). These results suggest that the identified variant in the pore region of Cav3.2 allows greater Ca^2+^ influx into NPCs derived from patient F2688-1, which is consistent with the enlarged pore size predicted by the 3D-model structure of the mutant channel.

Next, we sought to verify whether the mTORC1 signaling overactivation previously observed in F2688-1 NPCs [14] is due to greater influx of Ca^2+^ into cells via the mutant Cav3.2 channel. We treated F2688-1 and control NPCs with NNC 55-0396 and analyzed the phosphorylation status of RPS6, a downstream target of the mTORC1 pathway. We observed that whereas vehicle-treated F2688-1 NPCs showed, as expected, elevated levels of pRPS6, the blockade of T-type Ca^2+^ channels significantly reduced pRPS6 expression in F2688-1 NPCs to levels similar to the untreated control NPCs (Fig. 3A). These results suggest that the enhanced Ca^2+^ entry into F2688-1 NPCs through the mutant Cav3.2 channel causes excessive mTORC1 signaling activity in these cells.

### Mutant Cav3.2-mediated increase in mTORC1 signaling contributes to impaired Reelin signaling in F2688-1 neural progenitor cells

To test the hypothesis that the exaggerated mTORC1 signaling activation due to increased influx of Ca^2+^ through the mutant Cav3.2 channel in F2688-1 NPCs contributes to the impaired Reelin signaling in these cells, we treated F2688-1 and control-derived NPCs with NNC 55-0396 and analyzed the phosphorylation levels of SRC and DAB1, downstream targets of Reelin [37, 38]. We found that whereas vehicle-treated F2688-1 NPCs showed, as expected, decreased expression of pSRC and pDAB1 [14], the T-type Ca^2+^ channels blocker significantly enhanced pSRC expression to levels similar to the untreated control NPCs (Fig. 3B). On the other hand, while the T-type Ca^2+^ channels blocker significantly improved pDab1 levels in F2688-1 NPCs, there is still a clear trend towards diminished expression of pDAB1 in these cells compared to untreated control cells (Fig. 3B), corroborating our previous findings indicating that the variants in the *RELN* gene contribute to defective levels of this key regulator of Reelin signaling [14]. These results support our hypothesis of an abnormal interaction between mutated *CACNA1H* and *RELN* genes, showing that the mutant Cav3.2 further exacerbates impaired Reelin signaling in F2688-1 NPCs through the overactivation of mTORC1 pathway.

### Exaggerated Cav3.2/mTORC1 signaling causes enhanced proliferation of F2688-1 neural progenitor cells

**Fig. 4:**
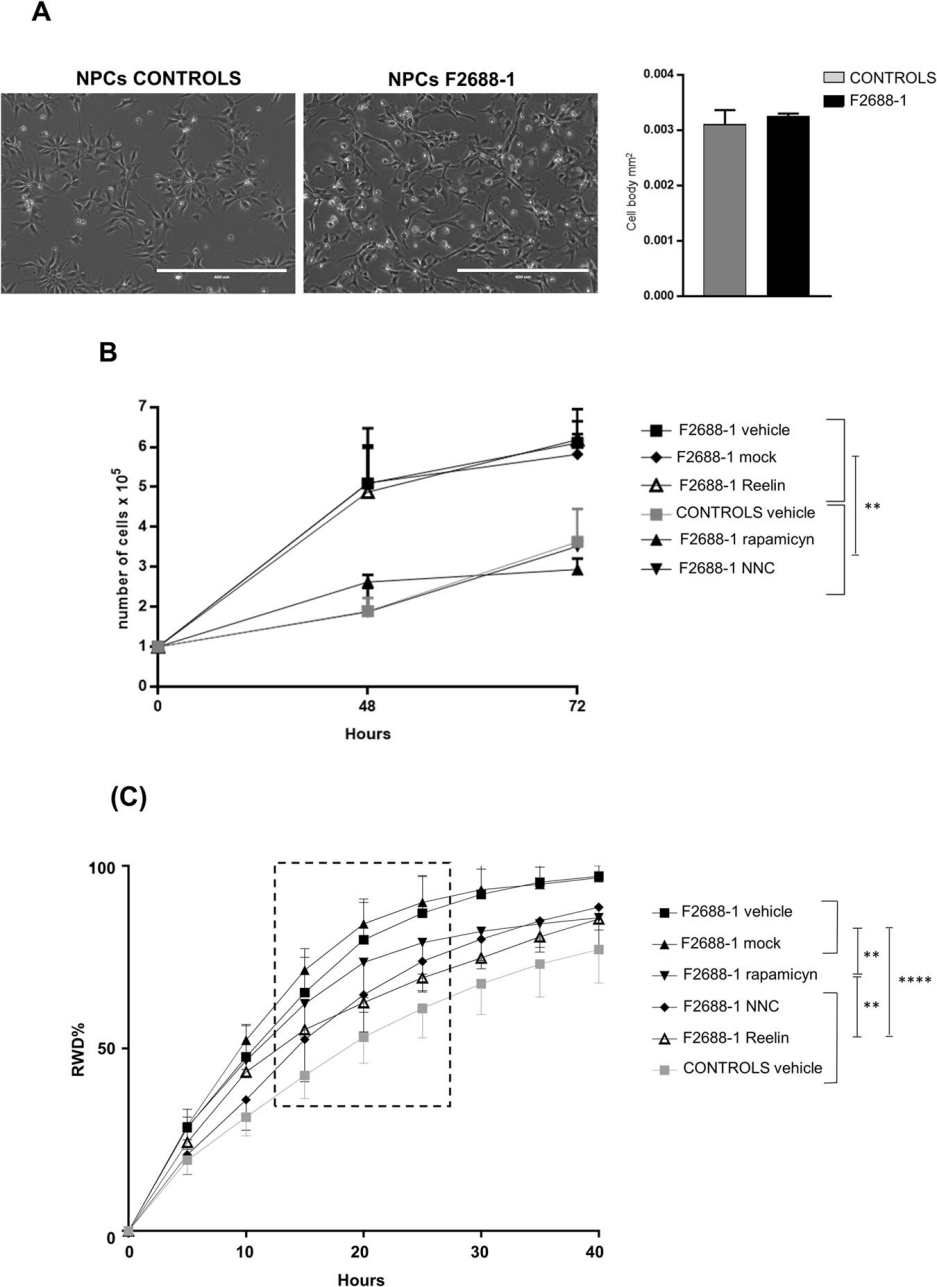
Mutant Cav3.2-mediated hyperfunction of mTORC1 signaling acts alone to cause enhanced proliferation of F2688-1-derived NPCs and together with impaired Reelin signaling in the abnormal migration of these cells. **A** Representative phase-contrast microscopy photographs of control- and F2688-1-derived NPCs. The bar graph shows the median cell body size (including soma size and all cell surface projections) of control-derived NPCs (*n* = 4) and F2688-1-derived NPCs (NPCs derived from 2 iPSC clones). There were no significant differences in median body size between the groups. **B** Line graph showing cell proliferation curves (live cell numbers at Day 0, 48 h, 72 h) of control-derived NPCs (*n* = 4) and F2688-1-derived (NPCs derived from 2 iPSC clones) cultured in the presence of either vehicle, 1 μM of NNC 55-0396 (NNC), or 100 nM of rapamycin; and in the presence of either mock-conditioned medium (mock) or Reelin-conditioned medium (Reelin). The graph shows the median value and interquartile range for each group. F2688-1 NPCs show significantly higher proliferation rates compared with control NPCs, which were completely rescued by treatment with either rapamycin or NNC. On the other hand, treatment with Reelin had no effect on the proliferation of F2688-1 NPCs. **C** Line graph showing the percentage relative wound density (RWD) over time in control-derived NPCs (*n* = 4) and in F2688-1-derived NPCs (NPCs derived from 2 iPSC clones) cultured in the presence of either vehicle, 1 μM of NNC, or 100 nM of rapamycin; and in the presence of either mock or Reelin. The graph shows the median value and interquartile range for each group. F2688-1 NPCs exhibit significantly higher migration rates compared with control NPCs, which was significantly attenuated by treatment with rapamycin, and completely rescued to levels equal to those of the untreated control NPCs in the exponential phase of the healing curve by treatment with either NNC or Reelin (analyzed time interval: 15 h–25 h). **p ≤ 0.01, ****p ≤ 0.0001.

Hyperactivation of mTORC1 signaling has been associated with abnormal morphology, size, and number of neural cells [39–43]. Therefore, we sought to determine whether the enhanced Cav3.2/mTORC1 signaling in F2688-1 NPCs, which in turn further aggravates Reelin signaling impairment, leads to changes in the morphology, size, and proliferation rates of these cells. While no gross alterations in cell morphology and no significant differences in cell body size (including soma size and all cell surface projections) were observed between control- and F2688-1-derived NPCs (Fig. 4A), F2688-1 NPCs show significantly higher proliferation rates compared with control NPCs, which were completely rescued by treatment with either the mTOR inhibitor rapamycin or NNC 55-0396. On the other hand, treatment with wild-type Reelin had no effect on the proliferation of F2688-1 NPCs (Fig. 4B). Since we have previously demonstrated that treatment of F2688-1 NPCs with wild-type Reelin significantly improved the defective Reelin pathway [14], these results suggest that mutant Cav3.2-mediated hyperfunction of mTORC1 signaling acts independently of the defective Reelin signaling to cause enhanced proliferation of F2688-1 NPCs.

### Abnormal Cav3.2 and Reelin induce aberrant migration of F2688-1 neural progenitor cells

Impaired Reelin signaling due to constitutive activation of the mTORC1 pathway plays an important role in abnormal neuronal migration in tuberous sclerosis complex (TSC) pathology [44]. Therefore, we next sought to investigate whether the mutant Cav3.2-mediated overactivation of mTORC1 signaling and reduction of Reelin signaling could affect the migration pattern of F2688-1 NPCs. Using the wound healing assay, we observed that the NPCs polarize toward the wound, extend filopodia protrusions, migrate, and closure the wound (Fig. S2). Also, we found that F2688-1 NPCs show significantly higher migration rates compared with control NPCs, which were significantly attenuated by treatment with rapamycin and completely rescued to levels equal to those of the untreated control NPCs in the exponential phase of the healing curve by treatment with either NNC 55-0396 or wild-type Reelin (Fig. 4C). Since we have previously shown that treatment of F2688-1 NPCs with rapamycin or wild-type Reelin led to a significant improvement in Reelin signaling deficits [14], these findings suggest that altered Cav3.2/mTORC1/Reelin pathways are all involved in the abnormal migration of F2688-1 NPCs.

### Heterologous expression of mutant Cav3.2 channel confirms its functional relevance

**Fig. 5:**
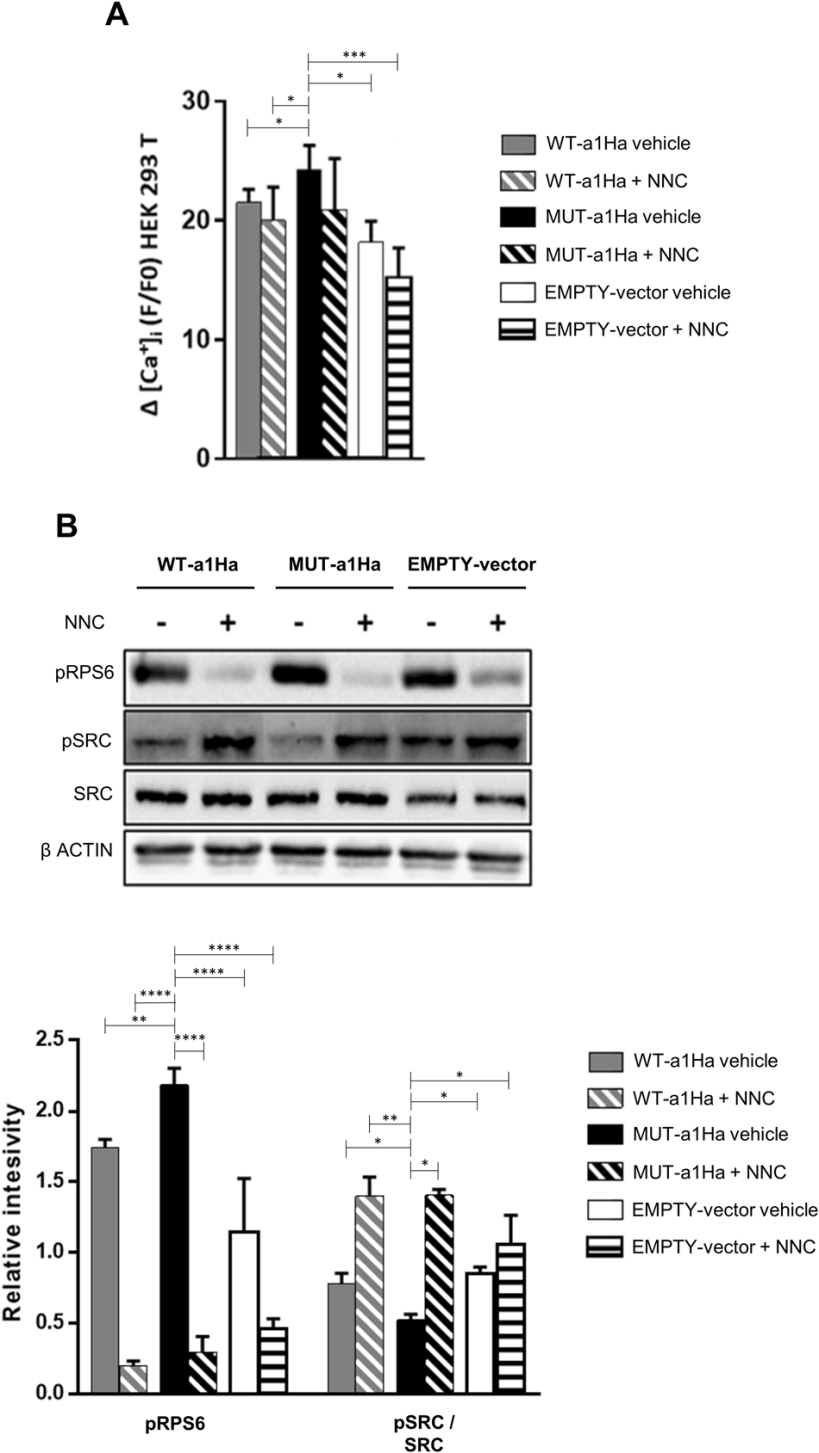
HEK293T cells overexpressing mutant Cav3.2 channel show increased Ca^2+^ influx, hyperfunctional mTORC1 signaling, and impaired activation of a Reelin signaling component. HEK293T cells were transfected with an empty vector (EMPTY-vector; *n* = 5) or with plasmids expressing either wild-type (WT-a1Ha; *n* = 5) or mutant (MUT-a1Ha; *n* = 5) Cav3.2 channels. **A** Intracellular Ca^2+^ measurements in transfected cells depolarized with 100 mM KCl and cultured in the absence (vehicle) or presence of 10 μM of the T-type Ca^2+^ channels blocker NNC 55-0396 (NNC). The bar graph shows the median value and interquartile range for each group. Cells overexpressing mutant Cav3.2 show significantly increased Ca^2+^ influx, which was rescued by treatment with NNC. Only the *p*-values obtained from the analyses using MUT-a1Ha are shown. **B** Representative immunoblots showing the expression levels of pRPS6, pSRC, SRC, and β-actin (used as loading control) in transfected cells cultured in the absence (−) or presence (+) of 10 μM of NNC. The bar graph shows the median levels and interquartile ranges of normalized pRPS6 and pSRC for each group; only the *p*-values obtained from the analyses using MUT-a1Ha are shown. Cells overexpressing mutant Cav3.2 show significantly increased pRPS6 levels and significantly decreased pSRC levels, which was rescued by treatment with NNC. *p < 0.05, **p ≤ 0.01, ****p ≤ 0.0001.

To provide additional support for our results, we next examined whether mutant Cav3.2 channel when heterologously expressed in HEK293T cells would alter Ca^2+^ influx and affect endogenous mTORC1 and Reelin pathway components. To this end, HEK293T cells were transfected with an empty pcDNA3 vector or with plasmids expressing either wild-type (WT-a1Ha) or mutant (MUT-a1Ha) Cav3.2 channels, and the influx of extracellular Ca^2+^ and the phosphorylation status of RPS6 and SRC were measured (Dab1 is not endogenously expressed in HEK293T cells). We observed that cells overexpressing both wild-type and mutant Cav3.2 channels show significantly increased Ca^2+^ influx after depolarization (Fig. 5A) and significantly augmented pRPS6 levels (Fig. 5B) compared with cells overexpressing the empty vector, suggesting that both the amount of Ca^2+^ ions entering the cells and the activity of the mTORC1 pathway are dependent on the number of Cav3.2 channels expressed in the plasma membrane. Also, in accordance with the results using F2688-1 NPCs, we observed that Ca^2+^ entry and pRPS6 levels were significantly greater in cells overexpressing mutant Cav3.2, and that treatment with NNC 55-0396 rescued these phenotypes (Fig. 5B). Finally, we observed that cells overexpressing mutant Cav3.2 show significantly decreased pSRC levels compared with cells overexpressing both the wild-type Cav3.2 and the empty vector, and that treatment with NNC 55-0396 also rescued this phenotype (Fig. 5B).

Taken together, these results strongly support that the variant identified in *CACNA1H* in patient F2688-1 exerts a gain-of-function effect by enhancing Ca^2+^ influx through the mutant Cav3.2 channel, which overactivates mTORC1 signaling and, in turn, contributes to impaired activation of Reelin signaling components in these cells.

### Increased burden of co-occurring risk variants in both alleles of Reelin pathway genes and in one allele of Ca^2+^ channel genes in ASD individuals

We hypothesized that the concomitant occurrence of rare deleterious variants (MAF ≤ 0.01; CADD score ≥20) in both alleles of genes for the Reelin cascade and in at least one allele of genes for Ca^2+^ channels may also be observed in other ASD individuals. To test this hypothesis, we initially analyzed the WES results from our Brazilian cohort of 279 trio and 6 quartet families with ASD (*n* = 291 individuals with ASD, 570 unaffected parents), and identified in two additional probands, referred to as F10832-1 and F11463-1, this specific combination of hits (Table 1; Supplementary Table S2; Supplementary Fig. S1B, C). Proband F10832-1 carries rare compound heterozygous variants in *VLDLR*, which encodes one of the receptors for Reelin [45–47], and a rare heterozygous variant in *CACNA2D4*, which encodes the voltage-gated L-type Ca^2+^ channel auxiliary subunit of the α2δ4 type [48–50]. Proband F11463-1 harbors rare compound heterozygous variants in *RELN*, and a rare heterozygous variant in *RYR3*, which encodes the intracellular Ca^2+^ release channel ryanodine receptor 3 [51–53]. Importantly, in silico analysis using several pathogenicity prediction tools strongly suggested that these variants are deleterious (Supplementary Table S2), and this particular combination of rare putatively damaging variants was not found in the parents, suggesting a significant enrichment of such combination in ASD individuals (*p* = 0.04). It is also noteworthy that it was not observed in the probands a significant excess of other combinations of risk variants in these genes, such as compound heterozygous variants in genes for the Reelin pathway alone (without co-occurring variants in Ca^2+^ channel genes), or variants in Ca^2+^ channel genes alone (without co-occurring variants in Reelin cascade genes), or two in cis variants in a Reelin pathway gene with co-occurring variants in Ca^2+^ channel genes, or heterozygous variants in a Reelin pathway gene with co-occurring variants in Ca^2+^ channel genes.

**Table 1:** Rare variants identified in both alleles of either *RELN* or *VLDLR* genes and in one allele of genes for Ca^2+^ channels in individuals with ASD.

Patient ID	Gene	Region	Variant description	Relation	Inheritance
Brazilian cohort					
F2688-1	*RELN*	Exonic	RELN:NM_005045:exon48:c.C7538G:p.S2513C	Proband	Father
	*RELN*	Exonic	RELN:NM_005045:exon48:c.C7634T:p.A2545V		Mother
	*CACNA1H*	Splicing	CACNA1H:NM_021098:exon13:c.2907 + 1G>A		De novo
F10832-1	*VLDLR*	Exonic	VLDLR:NM_003383:exon8:c.T1132C:p.Y378H	Proband	Father
	*VLDLR*	Exonic	VLDLR:NM_003383:exon13:c.G1901A:p.R634H		Mother
	*CACNA2D4*	Exonic	CACNA2D4:NM_172364:exon22:c.C2095T:p.L699F		Father
F11463-1	*RELN*	Exonic; Splicing	RELN:NM_005045:exon38:c.C5618T:p.T1873I	Proband	Father
	*RELN*	Exonic	RELN:NM_005045:exon42:c.G6343A:p.G2115S		Mother
	*RYR3*	Exonic	RYR3:NM_001036:exon20:c.G2486A:p.R829H		Mother

MSSNG cohort					
1-1098-003	*RELN*	Exonic	RELN:NM_005045:exon39:c.G5961T:p.K1987N	Proband	Father/de novo
	*RELN*	Exonic	RELN:NM_005045:exon34:c.C5108G:p.P1703R		Mother
	*RYR2*	Exonic	RYR2:NM_001035:exon67:c.G9569A:p.R3190Q		Mother
2-1259-004	*RELN*	Exonic	RELN:NM_005045:exon54:c.C8795A:p.S2932Y	Affected sibling	NA
	*RELN*	Exonic	RELN:NM_005045:exon25:c.C3477A:p.N1159K		NA
	*RYR1*	Exonic	RYR1:NM_000540:exon29:c.C4213A:p.P1405T		NA
5-5057-003	*RELN*	Exonic	RELN:NM_005045:exon20:c.G2689A:p.D897N	Proband	Father
	*RELN*	Exonic	RELN:NM_005045:exon34:c.C5108G:p.P1703R		Mother
	*ORAI1*	Exonic	ORAI1:NM_032790:exon2:c.126_127insA:p.A42fs		Mother
7-0276-003	*VLDLR*	Exonic	VLDLR:NM_003383:exon14:c.G1967A:p.R656H	Proband	Father/de novo
	*VLDLR*	Exonic	VLDLR:NM_003383:exon10:c.G1313A:p.G438D		Father/de novo
	*VLDLR*	Exonic	VLDLR:NM_003383:exon3:c.A242G:p.N81S		Mother
	*CACNA1B*	Exonic	CACNA1B:NM_000718:exon21:c.A3370G:p.I1124V		Mother
AU2168301	*RELN*	Exonic	RELN:NM_005045:exon2:c.T334C:p.F112L	Proband	Father
	*RELN*	Exonic	RELN:NM_005045:exon34:c.C5108G:p.P1703R		Mother
	*GRIN2C*	Exonic	GRIN2C:NM_000835:exon2:c.C193T:p.L65F		Mother
AU3756301	*RELN*	Exonic	RELN:NM_005045:exon39:c.G5923A:p.G1975S	Proband	Father/de novo
	*RELN*	Exonic	RELN:NM_005045:exon48:c.C7580A:p.S2527Y		Mother
	*CACNA2D4*	Exonic	CACNA2D4:NM_172364:exon37:c.G3245A:p.C1082Y		Father/de novo
	*CACNA1H*	Exonic	CACNA1H:NM_021098:exon9:c.G1508A:p.R503H		Mother
AU4027306	*RELN*	Exonic	RELN:NM_005045:exon1:c.C59T:p.T20M	Affected sibling	Father
	*RELN*	Exonic	RELN:NM_005045:exon42:c.G6458A:p.G2153D		Mother
	*CACNA1A*	Exonic	CACNA1A:NM_001127222:exon46:c.C6772A:p.H2258N		De novo
	*CACNB2*	Exonic	CACNB2:NM_201571:exon14:c.C1891T:p.R631C		Father

In order to further strengthen our findings, we next analyzed the WGS data from a large and independent cohort of ASD cases, the MSSNG resource [3], presently consisting of 11,181 samples from families with ASD, including 5102 ASD subjects. We found that 7 ASD individuals carry compound heterozygous risk variants in either the *RELN* or the *VLDLR* gene and also a risk variant in at least one Ca^2+^ channel gene, including *RYR2* [54, 55]; *RYR1* [56]*, ORAI1* [57]*, CACNA1B* [58]; *GRIN2C* [59]*, CACNA1A* [60], *CACNB2* [61]*, CACNA1H* [21]*, CACNA2D4* [48–50] (Table 1; Supplementary Table S2; Supplementary Fig. S1D–J). This specific combination of risk variants was not found in 6079 unaffected parents or siblings, and different combinations of risk variants in these genes were not found to be significantly enriched in the ASD subjects as observed in the Brazilian cohort, which provides corroborating evidence for a significantly increased burden of co-occurring impactful variants in both copies of either the *RELN* or the *VLDLR* gene and in one copy of Ca^2+^ channel genes in ASD (*p* = 0.004). Finally, it is also noteworthy that the ASD subjects from both the Brazilian and the MSSNG cohorts who harbor this specific combination of variants do not carry rare variants that cause a known deleterious loss of function (LoF) of a high-penetrant ASD gene. However, some of these individuals also carry risk variants in genes previously shown to contribute to ASD with reduced penetrance: the affected sisters 2-1259-003 and 2-1259-004 harbor a CNV disrupting the *ELP4* gene [62] (Supplementary Fig. S1E), and the proband AU2168301 carries a *de novo* LoF variant in the *KDM6B* gene [63] (Supplementary Fig. S1H).

## Discussion

Identifying the combinations of genetic variants required for determining ASD causality will aid in the understanding of the complex genetic architecture of ASD and of disease pathophysiology. In this study, we describe the functional analysis of a *de novo* splice site variant identified in the *CACNA1H* gene in an individual with ASD and macrocephaly who also carries deleterious compound heterozygous missense variants in the *RELN* gene [14]. We found that the variant in *CACNA1H* leads to the retention of an intronic sequence within the mature transcript, which is predicted to cause an in-frame insertion of 52 novel amino acid residues in the pore-forming domain of the Cav3.2 channel. The 3D model-structure of the mutated channel suggested that the pore may be enlarged in size, and we showed that Ca^2+^ influx through the mutant Cav3.2 is increased in patient-derived NPCs, which overactivates mTORC1 pathway and, consequently, further aggravates the impairment of Reelin signaling. Heterologous expression of the mutated Cav3.2 channel confirmed these results.

Within the CNS, Cav3.2 is predominantly expressed in the hippocampus, and Cav3.2 knockout mice show some behavioral abnormalities reminiscent of human autism [64, 65]. Rare missense variants in the human Cav3.2 have previously been reported in ASD individuals and appear to contribute to ASD susceptibility [21, 66–68]. Electrophysiological analysis of some of these variants using a non-neuronal exogenous expression system revealed that mutated Cav3.2 channels conducted substantially less current than wild-type channels. However, two of these variants located within the voltage-sensing region of Cav3.2 were shown to decrease the inactivation rates of the mutated channels and, thus, are expected to allow larger Ca^2+^ influx once activated [21], an effect similar to the variant here described. Therefore, a detailed biophysical analysis investigating the effects of ASD-associated Cav3.2 variants in more native conditions are still necessary to understand their characteristics and functional impact on neuronal excitability and function.

It should also be noted that common and rare variants in other Ca^2+^ channel genes (*CACNA1A*, *CACNA1C, CACNA1G*, *CACNA1I*, *CACNA1D*, *CACNA2D3*, and *CACNB2*) have been found to be associated with ASD [59, 60, 67–73], and functional analysis of some of these variants showed either gain- or loss-of-function of channel activity [71, 73]. Altogether, these findings suggest that perturbed intracellular Ca^2+^ homeostasis due to voltage-gated Ca^2+^ channel dysfunctions may affect neurodevelopmental processes contributing to ASD. In this sense, it is possible that the enhanced proliferation of F2688-1 NPCs caused by aberrant Cav3.2/Ca^2+^-mediated increase in mTORC1 signaling, which in turn further exacerbates the Reelin signal transduction defect and leads to abnormal migration of these cells might contribute to the macrocephaly and ASD symptoms observed in the patient. It has been shown that Ca^2+^ and mTORC1 pathways regulate neural cell proliferation [74, 75] and that hyperactive mTORC1 signaling is associated with enlarged head/brain size [24, 76], which is commonly observed in children with ASD [77]. It is also noteworthy that several neuropathological findings in the brains from autistic subjects support neuronal migration abnormalities [78], and that, in addition to the TSC mouse model that shows mTORC1/Reelin-mediated deficits in neuronal migration [44], aberrant neuronal migration have also been observed in many rodent genetic models of ASD that mimic disease-causing mutations in patients [79–81].

Analysis of WES data from our Brazilian cohort of 285 families with ASD and the analysis of WGS data from the MSSNG cohort of 4,258 families with ASD suggested that the co-occurrence of rare and potentially damaging variants in both alleles of either the *RELN* or the *VLDLR* gene and in one allele of Ca^2+^ channel genes confer significant liability for ASD. The existence of an oligogenic inheritance of ASD has been unrevealed by a series of studies [2, 10, 82–85], and recent analyses using large scale genomic data have suggested that genes involved in this multi-hit model show higher functional connectivity [12, 13, 86]. The data reported here corroborate these results, lending further support to the concept that genes with co-occurring deleterious variants tend to have interconnected signaling pathways.

## Supplementary information

Supplementary information

Table S1

Table S2

Table S3

Fig. S1

Fig. S1_continued

Fig. S2
