# Supplementary material for: Rare CACNA1H and RELN variants interact through mTORC1 pathway in oligogenic autism spectrum disorder
Source: Transl Psychiatry. 2022 Jun 6;12:234. doi: 10.1038/s41398-022-01997-9 (PMC9170683; doi:10.1038/s41398-022-01997-9)
Supplement: Supplementary file 1 — Supplementary information [file 41398_2022_1997_MOESM1_ESM.docx]

**Supplementary information**

**Whole-exome sequencing and data processing.** The Brazilian cohort consists of 279 trio and 6 quartet families of children diagnosed with ASD (n= 291 individuals with ASD, 570 unaffected parents). All affected individuals were diagnosed by psychiatrists using the criteria defined in DSM-V, and CARS and/or interviews based on ADI-R and/or ADOS were applied whenever possible. Exome-sequencing libraries using genomic DNA from peripheral blood were generated as previously described [4, 14-16] and were sequenced on the Illumina HiSeq 2500 system. Sequence alignments to the human reference genome (build GRCh38/hg38) were performed with Burrows–Wheeler Aligner (BWA; bwakit version 0.7.15), data processing and variant calling were performed using Genome Analysis Toolkit package (GATK; version 4.1.4.). An in-house-script was used to split multiallelic variants and BCFtools (version 1.7) was used to left-alignment. All the variants were annotated with both ANNOVAR and an in-house-script and were visually inspected using Integrative Genomics Viewer software. Variants with a minimum of 20 reads for heterozygotes and 10 reads for homozygotes were selected, as well as allele balance between 30% and 70%. Global minor allele frequency (MAF) was checked on public databases as The Genome Aggregation Database (gnomAD_all), 1,000 Genomes Project (1000G), Exome Sequencing Project v.6500 (ESP6500) and the Brazilian database ABraOM. Based on the deleteriousness prediction scores for the *RELN* variants identified in the F2688-1 proband [14], deleteriousness prediction of rare variants (MAF ≤0.01) was initially based on CADD_phred score ≥20. Co-occurring rare variants with CADD_phred score ≥20 in both alleles of Reelin pathway genes and in one allele of Ca^2+^ channel genes were validated by Sanger sequencing and were also tested with SIFT, Polyphen2_HDIV, Polyphen2_HVAR, LRT, MutationTaster, MutationAssessor, FATHMM, PROVEAN, MetaSVM, MetaLR, M-CAP, fathmm-MKL_coding and Human Splicing Finder. We considered ASD genes whenever present on the SFARI database (https://gene.sfari.org/).

**Whole-genome sequencing and data processing.** the MSSNG resource analyzed (https://research.mss.ng) consists of 11,181 individuals from 4,258 unique families with children diagnosed with ASD, including 3,913 probands, 1,178 affected siblings and 11 additional family members (n= 5,102 ASD individuals). Genome-sequencing libraries using genomic DNA from peripheral blood or lymphoblast-derived cell lines were prepared as previously described [3] and were sequenced using Complete Genomics, Illumina HiSeq 2000 and HiSeq X technology. Sequencing reads were aligned to human genome build 37 (GRCh37/hg19) using the BWA (version 0.7.10). Picard (version 1.117) was used for sorting by chromosome coordinates and marking duplicates. Local realignment and quality recalibration was performed with GATK (version 3.3) on each chromosome. Single SNVs and indels were detected using GATK with HaplotypeCaller. Nonvariant segments (reference intervals) that were emitted by HaplotypeCaller were extracted using a custom Java program (NonVariantSiteFilter.jar). Variants were required to have genotype quality scores (GQ for Illumina; VAF for Complete Genomics) of at least 99. Based on the same selection criteria as for the Brazilian cohort, variants in genes for the Reelin pathway and Ca^2+^ channels (Supplementary Table S1) with MAF ≤0.01 in public databases (gnomAD, 1,000G, ESP6500 and ABraOM) and with CADD_phred score ≥20 were selected. Subsequently, co-occurring rare variants with CADD_phred score ≥20 in both alleles of Reelin pathway genes and in one allele of Ca^2+^ channel genes were validated by Sanger sequencing and were tested with the same 13 pathogenic prediction tools mentioned above.

**Supplementary Table S1.** List of genes screened for the presence of co-occurring rare and potentially protein-damaging variants in individuals with ASD.

**Supplementary Table S2.** Extended description of the rare variants in both alleles of either *RELN* or *VLDLR* genes and in one allele of genes for Ca^2+^ channels in individuals with ASD.

**Supplementary Table S3.** Clinical characteristics of the ASD subjects who carry risk variants in both alleles of either *RELN* or *VLDLR* genes and in one allele of Ca^2+^ channel genes.

**Supplementary Fig. 1S. Pedigrees of families in whom co-occurring risk variants in both copies of either *RELN* or *VLDLR* genes and in one copy of Ca^2+^ channel genes segregate with ASD. A-C** Pedigrees of the Brazilian families; **D-J** Pedigrees of the MSSNG families. Open symbols indicate healthy individuals and solid black symbols indicate affected individuals. The individual identification numbers and the variants found in the *RELN* and *VLDLR* genes as well as in the Ca^2+^ channel genes are indicated. Importantly, these ASD subjects do not carry other rare variants that cause a known deleterious LoF of a high-penetrant ASD gene. However, the affected sisters 2-1259-003 and 2-1259-004 harbor a CNV disrupting the *ELP4* gene, and the proband AU2168301 carries a *de novo* LoF variant in the *KDM6B* gene, which were previously shown to contribute to ASD with incomplete penetrance.

**Supplementary Fig. 2S. Scratch wound healing assay.** Representative phase-contrast microscopy photographs of control- and F2688-1-derived NPCs showing that cells at the wound edges polarize toward the wound, extend filopodia protrusions, and migrate.
