# Supplementary material for: Rare CACNA1H and RELN variants interact through mTORC1 pathway in oligogenic autism spectrum disorder
Source: Transl Psychiatry. 2022 Jun 6;12:234. doi: 10.1038/s41398-022-01997-9 (PMC9170683; doi:10.1038/s41398-022-01997-9)
Supplement: Supplementary file 2 — Table S1 [file 41398_2022_1997_MOESM2_ESM.docx]

**Table S1:** List of genes screened for the presence of co-occurring rare and potentially protein-damaging variants in individuals with ASD.

| **Calcium channel genes** | | | | |  | **Reelin pathway genes** | | |
| --- | --- | --- | --- | --- | --- | --- | --- | --- |
| *CACNA1A* | *CACNA2D1* | *CACNG2* | *RYR1* | *CASK* |  | *DAB1* | *RELN* | *CRK* |
| *CACNA1B* | *CACNA2D2* | *CACNG3* | *RYR2* | *GRIN1* |  | *FYN* | *SRC* | *NCK2* |
| *CACNA1C* | *CACNA2D3* | *CACNG4* | *RYR3* | *GRIN2A* |  | *ITGA3* | *VLDLR* | *RAP1A* |
| *CACNA1D* | *CACNA2D4* | *CACNG7* | *TPCN1* | *GRIN2B* |  | *LRP8* | *APOE* | *CUL5* |
| *CACNA1E* | *CACNB1* | *CACNG8* | *TPCN2* | *GRIN3A* |  |  |  |  |
| *CACNA1H* | *CACNB2* | *CACNB4* | *ORAI2* | *GRIN2D* |  |  |  |  |
| *CACNA1I* | *CACNB3* | *ORAI1* | *ORAI3* | *GRIN2C* |  |  |  |  |
