# Supplementary material for: Rare CACNA1H and RELN variants interact through mTORC1 pathway in oligogenic autism spectrum disorder
Source: Transl Psychiatry. 2022 Jun 6;12:234. doi: 10.1038/s41398-022-01997-9 (PMC9170683; doi:10.1038/s41398-022-01997-9)
Supplement: Supplementary file 3 — Table S2 [file 41398_2022_1997_MOESM3_ESM.docx]

**Table S2.** Extended description of the rare variants in both alleles of either RELN or VLDLR genes and in one allele of genes for Ca2+ channels identified in individuals with ASD.

**Brazilian cohort**

| **Patient ID** | **Gene** | **Region** | **Variant description** | **Relation** | **Inheritance** | **dbSNP** | **MAF GnomAD ^a^** | **MAF AbraOM ^b^** | **CADD score ^c^** | **Predicted to be pathogenic by ^d^** | **Splice site prediction ^e^** |
| --- | --- | --- | --- | --- | --- | --- | --- | --- | --- | --- | --- |
| F2688-1 | *RELN* | exonic | RELN:NM_005045:exon48:c.C7538G:p.S2513C | proband | Father | rs114647348 | 0.0008 | 0.0016 | 21.5 | 4 out of 12 prediction tools | No significant impact on splicing signals |
|  | *RELN* | exonic | RELN:NM_005045:exon48:c.C7634T:p.A2545V |  | Mother | rs116634494 | 0.0002 | 0.0008 | 25.2 | 7 out of 13 prediction tools | Significant alteration of ESE / ESS motifs ratio (-3) |
|  | *CACNA1H* | splicing | CACNA1H:NM_021098:exon13:c.2907+1G>A |  | De novo | Absent | 0 | 0 | 23.6 | 3 out of 3 prediction tools | Significant alteration of ESE / ESS motifs ratio (-8) |
| F10832-1 | *VLDLR* | exonic | VLDLR:NM_003383:exon8:c.T1132C:p.Y378H | proband | Father | rs142885301 | 0.0002 | 0.0016 | 25.4 | 11 out of 13 prediction tools | Significant alteration of ESE / ESS motifs ratio (-3) |
|  | *VLDLR* | exonic | VLDLR:NM_003383:exon13:c.G1901A:p.R634H |  | Mother | rs35339834 | 0.0007 | 0 | 35 | 12 out of 13 prediction tools | No significant impact on splicing signals |
|  | *CACNA2D4* | exonic | CACNA2D4:NM_172364:exon22:c.C2095T:p.L699F |  | Father | rs151121191 | 0.0016 | 0.0016 | 20.8 | 8 out of 13 prediction tools | Significant alteration of ESE / ESS motifs ratio (-2) |
| F11463-1 | *RELN* | exonic; splicing | RELN:NM_005045:exon38:c.C5618T:p.T1873I | proband | Father | rs41275239 | 0.0041 | 0.0049 | 22.6 | 5 out of 13 prediction tools | Significant alteration of ESE / ESS motifs ratio (-6) |
|  | *RELN* | exonic | RELN:NM_005045:exon42:c.G6343A:p.G2115S |  | Mother | rs116716038 | 0.0019 | 0.0074 | 23.3 | 5 out of 12 prediction tools | Significant alteration of ESE / ESS motifs ratio (-2) |
|  | *RYR3* | exonic | RYR3:NM_001036:exon20:c.G2486A:p.R829H |  | Mother | rs199500216 | 0.0011 | 0.0008 | 24.2 | 9 out of 12 prediction tools | No significant impact on splicing signals |

**MSSNG cohort**

| **Patient ID** | **Gene** | **Region** | **Variant description** | **Relation** | **Inheritance** | **dbSNP** | **MAF GnomAD ^a^** | **MAF AbraOM ^b^** | **CADD score ^c^** | **Predicted to be pathogenic by ^d^** | **Splice site prediction ^e^** |
| --- | --- | --- | --- | --- | --- | --- | --- | --- | --- | --- | --- |
| 1-1098-003 | *RELN* | exonic | RELN:NM_005045:exon39:c.G5961T:p.K1987N | proband | Father / de novo | rs143948239 | 0.000092 | 0 | 25.0 | 4 out of 13 prediction tools | No significant impact on splicing signals |
|  | *RELN* | exonic | RELN:NM_005045:exon34:c.C5108G:p.P1703R |  | Mother | rs2229860 | 0.0033 | 0 | 29.7 | 10 out of 13 prediction tools | Potential alteration of splicing |
|  | *RYR2* | exonic | RYR2:NM_001035:exon67:c.G9569A:p.R3190Q |  | Mother | rs369276868 | 0.000034 | 0 | 24.6 | 5 out of 13 prediction tools | No significant impact on splicing signals |
| 2-1259-004 | *RELN* | exonic | RELN:NM_005045:exon54:c.C8795A:p.S2932Y | affected sibling | NA | NA | 0.0000916 | 0 | 20.9 | 6 out of 13 prediction tools | No significant impact on splicing signals |
|  | *RELN* | exonic | RELN:NM_005045:exon25:c.C3477A:p.N1159K |  | NA | rs114684479 | 0.0016 | 0.0057 | 23.7 | 7 out of 13 prediction tools | No significant impact on splicing signals |
|  | *RYR1* | exonic | RYR1:NM_000540:exon29:c.C4213A:p.P1405T |  | NA | rs376567445 | 0.000016 | 0 | 20.3 | 10 out of 13 prediction tools | Significant alteration of ESE / ESS motifs ratio (-6) |
| 5-5057-003 | *RELN* | exonic | RELN:NM_005045:exon20:c.G2689A:p.D897N | proband | Father | rs115167821 | 0,000021 | 0 | 24,9 | 7 out of 13 prediction tools | No significant impact on splicing signals |
|  | *RELN* | exonic | RELN:NM_005045:exon34:c.C5108G:p.P1703R |  | Mother | rs2229860 | 0.0033 | 0 | 29.7 | 10 out of 13 prediction tools | Potential alteration of splicing² |
|  | *ORAI1* | exonic | ORAI1:NM_032790:exon2:c.126_127insA:p.A42fs |  | Mother | NA | 0 | 0.0032 | NA | NA | Significant alteration of ESE / ESS motifs ratio (-4) |
| 7-0276-003 | *VLDLR* | exonic | VLDLR:NM_003383:exon14:c.G1967A:p.R656H | proband | Father / de novo | NA | 0.0000119 | 0 | 26.1 | 12 out of 13 prediction tools | No significant impact on splicing signals |
|  | *VLDLR* | exonic | VLDLR:NM_003383:exon10:c.G1313A:p.G438D |  | Father / de novo | rs200605669 | 0.0004 | 0.0008 | 34 | 12 out of 13 prediction tools | No significant impact on splicing signals |
|  | *VLDLR* | exonic | VLDLR:NM_003383:exon3:c.A242G:p.N81S |  | Mother | rs140526335 | 0.0020 | 0.0032 | 21.8 | 7 out of 13 prediction tools | Significant alteration of ESE / ESS motifs ratio (6) |
|  | *CACNA1B* | exonic | CACNA1B:NM_000718:exon21:c.A3370G:p.I1124V |  | Mother | NA | 0.000012 | 0 | 20.2 | 3 out of 13 prediction tools | No significant impact on splicing signals |
| AU2168301 | *RELN* | exonic | RELN:NM_005045:exon2:c.T334C:p.F112L | proband | Father | NA | 0.0000159 | 0 | 23.4 | 8 out of 13 prediction tools | No significant impact on splicing signals |
|  | *RELN* | exonic | RELN:NM_005045:exon34:c.C5108G:p.P1703R |  | Mother | rs2229860 | 0.0033 | 0 | 29.7 | 10 out of 13 prediction tools | Potential alteration of splicing² |
|  | *GRIN2C* | exonic | GRIN2C:NM_000835:exon2:c.C193T:p.L65F |  | Mother | NA | 0.0050 | 0.0024 | 25.0 | 5 out of 13 prediction tools | Significant alteration of ESE / ESS motifs ratio (-3) |
| AU3756301 | *RELN* | exonic | RELN:NM_005045:exon39:c.G5923A:p.G1975S | proband | Father / de novo | rs114807343 | 0.0007 | 0.0032 | 26.2 | 8 out of 13 prediction tools | Significant alteration of ESE / ESS motifs ratio (-2)¹ |
|  | *RELN* | exonic | RELN:NM_005045:exon48:c.C7580A:p.S2527Y |  | Mother | rs114620403 | 0.000103 | 0 | 28.9 | 7 out of 13 prediction tools | No significant impact on splicing signals |
|  | *CACNA2D4* | exonic | CACNA2D4:NM_172364:exon37:c.G3245A:p.C1082Y |  | Father / de novo | NA | 0 | 0 | 28.1 | 13 out of 13 prediction tools | No significant impact on splicing signals |
|  | *CACNA1H* | exonic | CACNA1H:NM_021098:exon9:c.G1508A:p.R503H |  | Mother | rs201009269 | 0.000084 | 0 | 22.3 | 9 out of 13 prediction tools | No significant impact on splicing signals |
| AU4027306 | *RELN* | exonic | RELN:NM_005045:exon1:c.C59T:p.T20M | affected sibling | Father | rs145135688 | 0.000070 | 0 | 23 | 3 out of 13 prediction tools | No significant impact on splicing signals |
|  | *RELN* | exonic | RELN:NM_005045:exon42:c.G6458A:p.G2153D |  | Mother | rs144387303 | 0.000068 | 0.0008 | 27.4 | 9 out of 13 prediction tools | No significant impact on splicing signals |
|  | *CACNA1A* | exonic | CACNA1A:NM_001127222:exon46:c.C6772A:p.H2258N |  | De novo | NA | 0 | 0 | 22.6 | 8 out of 13 prediction tools | No significant impact on splicing signals |
|  | *CACNB2* | exonic | CACNB2:NM_201571:exon14:c.C1891T:p.R631C |  | Father | rs77141223 | 0.0002 | 0.0024 | 24.9 | 10 out of 13 prediction tools | Significant alteration of ESE / ESS motifs ratio (2)¹ |

| a = MAF GnomAD_exome_ALL |
| --- |
| b = MAF AbraOM (Online Archive of Brazilian Mutations) |
| c = CADD (Combined Annotation Dependent Depletion) threshold score of ≥20 |
| d = Missense variant prediction algorithms used: SIFT; Polyphen2_HDIV; Polyphen2_HVAR; LRT; MutationTaster; MutationAssessor; FATHMM; PROVEAN; MetaSVM; MetaLR; M-CAP; fathmm-MKL_coding; CADD_phred |
| e = Splice site prediction was performed using the Human Splicing Finder tool; ESE = exonic splicing enhancer; ESS = exonic splicing silencer. |
| 1 - Potential to activate a cryptic acceptor site |
| 2 - Potential to activate a cryptic donor site |
