# Supplementary material for: Rare CACNA1H and RELN variants interact through mTORC1 pathway in oligogenic autism spectrum disorder
Source: Transl Psychiatry. 2022 Jun 6;12:234. doi: 10.1038/s41398-022-01997-9 (PMC9170683; doi:10.1038/s41398-022-01997-9)
Supplement: Supplementary file 4 — Table S3 [file 41398_2022_1997_MOESM4_ESM.docx]

**Table S3:** Clinical characteristics of the ASD subjects who carry risk variants in both alleles of either *RELN* or *VLDLR* genes and in one allele of Ca^2+^ channel genes.

| **Brazilian cohort** | |  |
| --- | --- | --- |
| **Patient ID** | **Phenotype** | **Available tests** |
| **F2688-1** | ASD; CARS: 30.5; Socialization: low average; IQ: 110; macrocephaly (at 17 years old, OFC= 61 cm, >97th centile) | CARS, ADOS, WISC |
| **F10832-1** | ASD | NA |
| **F11463-1** | ASD; CARS: 32 | CARS |
|  |  |  |
| **MSSNG cohort** | |  |
| **Patient ID** | **Phenotype** | **Available tests** |
| **1-1098-003** | ASD | NA |
| **2-1259-004** | ASD; Global Ability: borderline; Adaptive Behaviour: severe; Socialization: severe; IQ: 75; allergies (treenut); asthma; little or no babble in 1^st^ year | ABAS, Vineland, SWAN, RCADS, ADI-R, ADOS, SCQ, SRS, ABC-C, CBCL, Leiter, CCC, OWLS, PPVT, Tanner, Eyes, RBS |
| **5-5057-003** | ASD | NA |
| **7-0276-003** | ASD | NA |
| **AU2168301** | ASD, Adaptive Behaviour: borderline; Socialization: low average | ADI-R, ADOS, Leiter, Griffiths, PPVT, body measurements |
| **AU3756301** | ASD, Global Ability: low average; IQ: 85 Raven Non-verbal IQ | ADI-R, SRS, Raven, PPVT |
| **AU4027306** | ASD, Global Ability: low average, IQ: 85 Raven Non-verbal IQ | ADI-R, SRS, Raven, PPVT |
