# Supplementary figures and images for: Rare CACNA1H and RELN variants interact through mTORC1 pathway in oligogenic autism spectrum disorder
Source: Transl Psychiatry. 2022 Jun 6;12:234. doi: 10.1038/s41398-022-01997-9 (PMC9170683; doi:10.1038/s41398-022-01997-9)

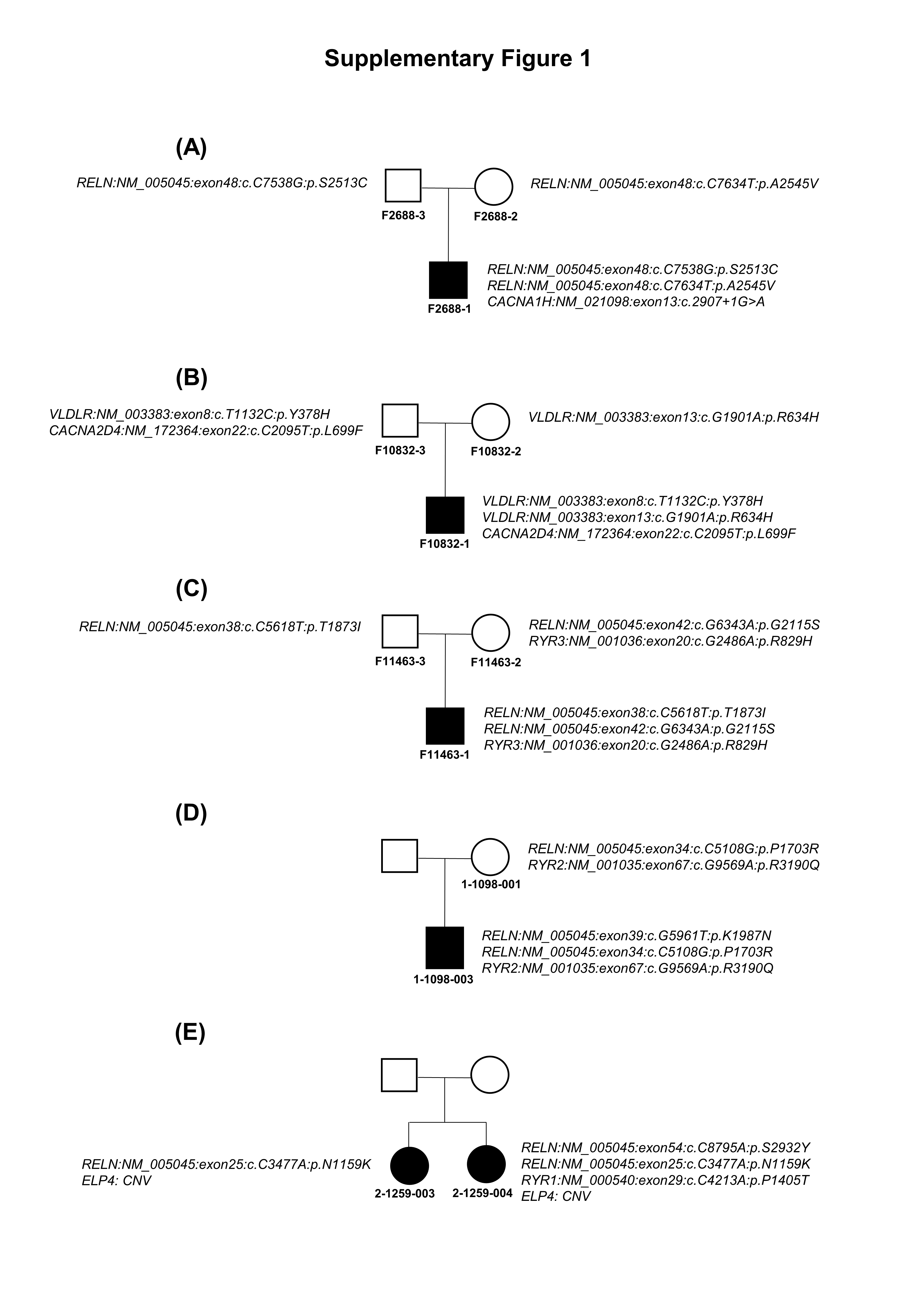

Supplement: Supplementary file 5 — Fig. S1 [file 41398_2022_1997_MOESM5_ESM.tif]

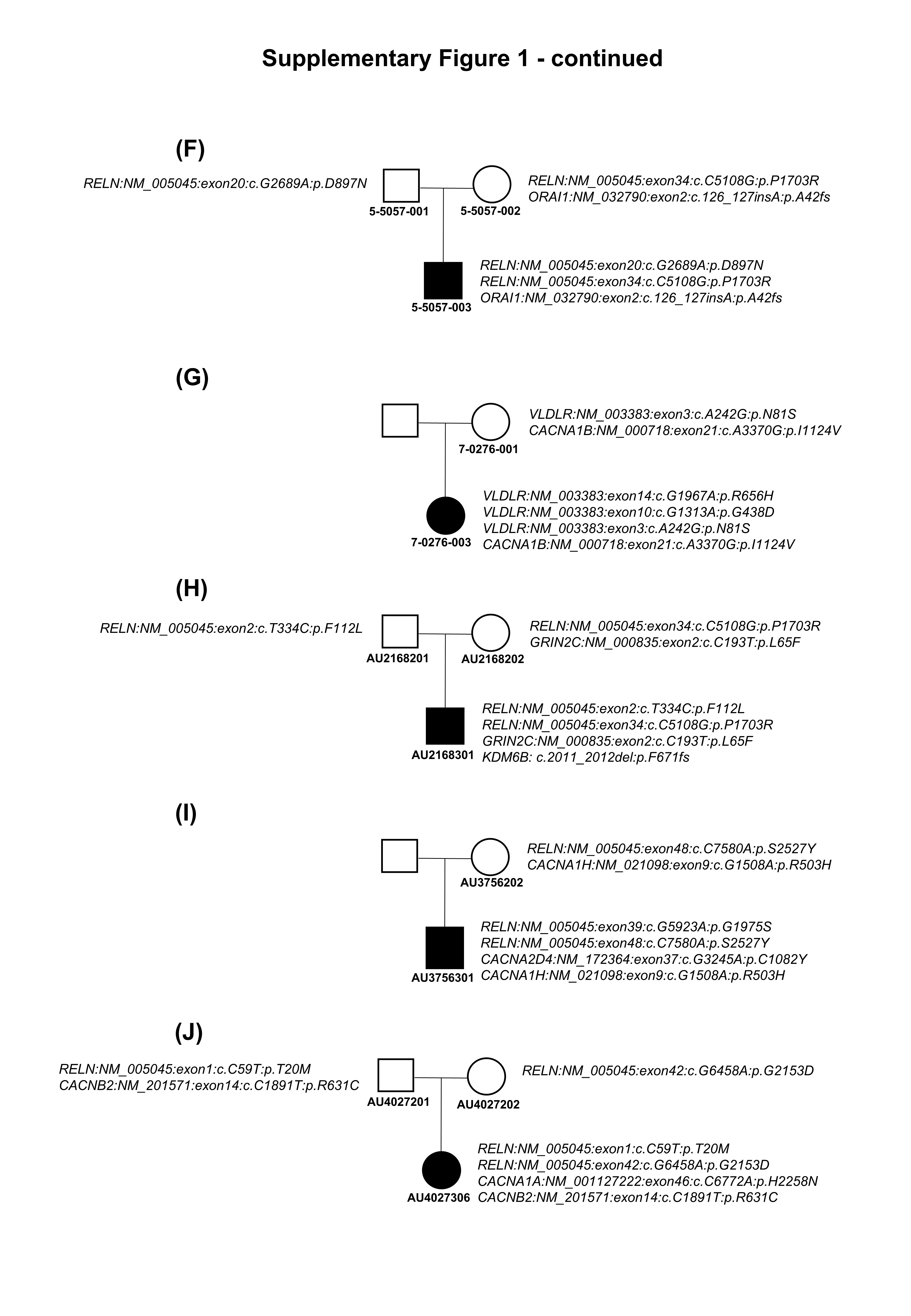

Supplement: Supplementary file 6 — Fig. S1_continued [file 41398_2022_1997_MOESM6_ESM.tif]

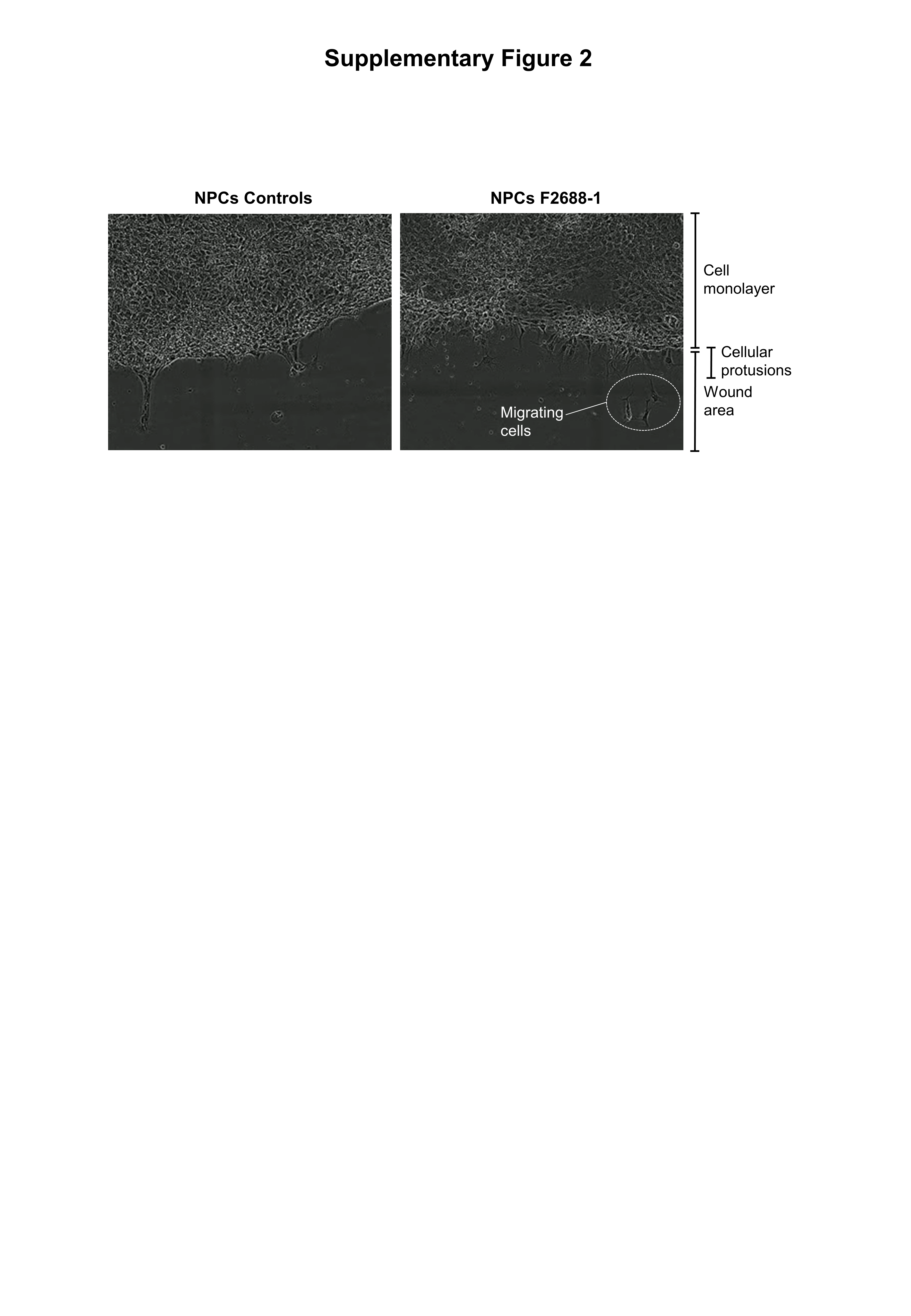

Supplement: Supplementary file 7 — Fig. S2 [file 41398_2022_1997_MOESM7_ESM.tif]
